# Tetracycline-inducible shRNA targeting long non-coding RNA PVT1 inhibits cell growth and induces apoptosis in bladder cancer cells

**DOI:** 10.18632/oncotarget.5880

**Published:** 2015-10-15

**Authors:** Chengle Zhuang, Jianfa Li, Yuchen Liu, Mingwei Chen, Jiancheng Yuan, Xing Fu, Yonghao Zhan, Li Liu, Junhao Lin, Qing Zhou, Wen Xu, Guoping Zhao, Zhiming Cai, Weiren Huang

**Affiliations:** ^1^ Key Laboratory of Medical Reprogramming Technology, Shenzhen Second People's Hospital, Shenzhen 518039, Guangdong Province, People's Republic of China; ^2^ Shantou University Medical College, Shantou 515041, Guangdong Province, People's Republic of China; ^3^ Anhui Medical University, Hefei 230601, Anhui Province, People's Republic of China; ^4^ Shanghai-MOST Key Laboratory of Health and Disease Genomics, Chinese National Human Genome Centerat Shanghai, Shanghai 200000, Shanghai, People's Republic of China

**Keywords:** PVT1, bladder cancer, lncRNAs, tetracycline-inducible, synthetic biology

## Abstract

Recent studies show that long non-coding RNAs (lncRNAs) may be significant functional regulators in tumor development, including bladder cancer. Here, we found that PVT1 was upregulated in bladder cancer tissues and cells. Further experiments revealed that PVT1 promoted cell proliferation and suppressed cell apoptosis. Furthermore we also used the emerging technology, synthetic biology, to create tetracycline-inducible small hairpin RNA (shRNA) vectors which silenced PVT1 in a dosage-dependent manner to inhibit the progression of bladder cancer. In conclusion, data suggest that PVT1 could be an oncogene and may be a therapeutic target in bladder cancer. Synthetic “tetracycline-on” switch system can be used to quantitatively control the expression of PVT1 in bladder cancer in response to different concentration of doxycycline to suppress the progression of bladder cancer.

## INTRODUCTION

Bladder cancer is one of the most common leading causes of cancer-related death in the world [1, 2]. Surgery, radiation therapy and chemotherapy are current major therapies for the treatment of patients diagnosed with bladder cancer but survival rate remains disappointed [3, 4]. One of the important reasons is lack of full understanding of the mechanism to treat bladder cancer. Recent studies suggest many oncogenes and tumor suppressors including long non-coding RNAs (lncRNAs) are key regulators for bladder cancer development. It indicates that lncRNAs may be novel indicators for treatment of bladder cancer.

Long non-coding RNAs (lncRNAs), which are greater than 200 nucleotides in length, involve in the development of various human diseases, especially in cancers [5–9]. PVT1 is upregulted in prostate cancer and associated with cancer development [10]. In ovarian cancer, PVT1 may be a vital downstream target for therapy [11]. Besides, PVT1 promotes progression of gastric cancer and hepatocellular carcinoma [12–14]. Furthermore, PVT1 overexpression acts independently of MYC to promote cell proliferation and suppress apoptosis in ovarian and breast cancer [15]. However, whether PVT1 also participates in development of bladder cancer is still unknown and needed to be studied.

Tetracycline-inducible system is the most widely utilized regulatory system and may be a useful tool in the emerging era of medical synthetic biology [16, 17]. The inducer, doxycycline, is nontoxic and used widely in preclinical and clinical studies [16]. It can control the expression of gene and may give us ideas to treat bladder cancer.

In this study, we found that PVT1 was upregulated in bladder cancer tissues and cells, and functioned as an oncogene in bladder cancer. shRNA sequences targeting PVT1 controlled by synthetic “tetracycline-on” switch suppressed the expression of PVT1 in response to different concentration of doxycycline and inhibited progression of bladder cancer.

## RESULTS

### PVT1 was upregulated in bladder cancer tissues, cells and its correlation with clinical pathologic factors

**Figure 1:**
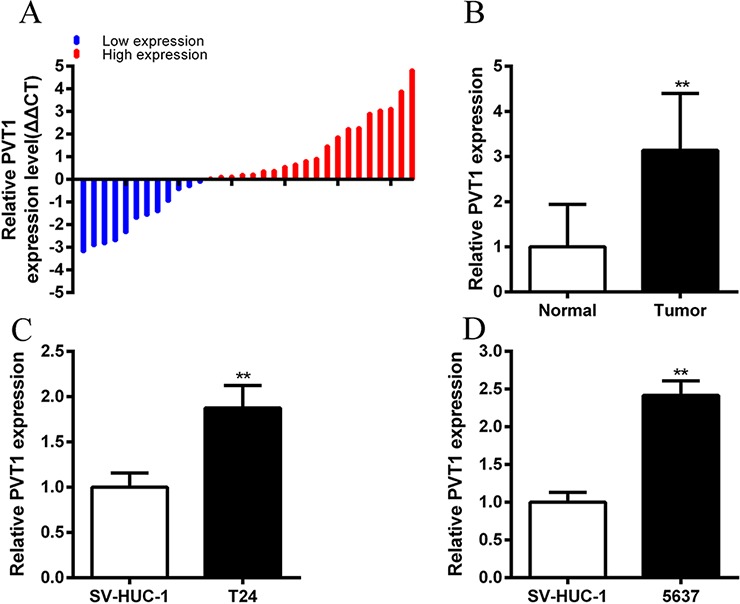
PVT1 was upregulated in bladder cancer tissues and cell lines **A.** qPCR was used to measure relative concentration of PVT1 in bladder cancer tissues. **B.** The relative expression level of PVT1 was significantly higher in bladder cancer tissues compared with matched normal tissues (*P* < 0.01). **C, D.** Compared with SV-HUC-1, the relative expression level of PVT1 was upregulated significantly in bladder cancer T24 (C) and 5637 (D) cells (*P* < 0.01). Error bars suggest mean ± SD (*P < 0.05, **P < 0.01).

The relative expression level of PVT1 was detected by performing real-time qPCR in a total of 32 patients with bladder cancer. Compared with normal counterparts, the PVT1 expression was upregulated significantly in 62.5% (20 of 32) of cancer tissues (*P* < 0.01) (Figure 1A and 1B). Compared with the SV-HUC-1 cell line, the PVT1 expression was increased significantly in bladder cancer cells, T24 (*P* < 0.01) and 5637 (*P* < 0.001) (Figure 1C and 1D). As Table 1 showed, upregulation of PVT1 was highly correlated with bladder cancer histological grade (*P* = 0.028). TNM stage also had a high association with upregulated expression of PVT1 (*P* = 0.002). But gender, age, tumor size and lymph node metastasis had no associations with PVT1 expression level. These data indicated that long non-coding PVT1 may function as an oncogene in bladder cancer. Patients' clinical parameters are listed in Table 2.

**Table 1:** Correlation between PVT1 expression and clinicopathological characteristics of bladder cancer patients

Parameters	Group	Total	PVT1 expression		*P* value
			High	Low	
Gender	Male	24	17	7	0.116
	Female	8	3	5	
Age (years)	<59	9	5	4	0.686
	≥59	23	15	8	
Tumor size (cm)	<3 cm	15	7	8	0.144
	≥3 cm	17	13	4	
Histological grade	L	18	8	10	0.028*
	H	14	12	2	
TNM stage	0/I	8	1	7	0.002*
	II/III/IV	24	19	5	
Lymph nodes metastasis	N0	29	19	10	0.540
	N1 or above	3	1	2	

* *P* < 0.05 was considered significant (Chi-square test between 2 groups).

**Table 2:** Summary of clinicopathological features of tissues of bladder cancer

Pt No.	Age	Sex	Grade	Stage	Pt No.	Age	Sex	Grade	Stage
1	53	M	L	T1N0M0	17	64	F	H	T2N0M0
2	61	F	H	T3N0M0	18	25	M	L	T1N0M0
3	75	M	H	T2N0M0	19	59	M	L	T2N0M0
4	57	M	H	T4N1M0	20	46	M	L	T1N0M0
5	62	M	H	T4N1M0	21	63	F	H	T3N0M0
6	65	M	H	T2N0M0	22	58	M	H	T1N0M0
7	64	M	H	T3N0M0	23	63	M	L	T2N0M0
8	63	M	H	T2N0M0	24	54	M	L	T2N0M0
9	70	M	L	T2N0M0	25	70	F	L	T1N0M0
10	59	M	L	T2N0M0	26	51	F	L	T1N0M0
11	73	F	L	T2N0M0	27	63	M	L	T2N0M0
12	68	M	L	T2N0M0	28	67	M	L	T2N0M0
13	50	M	H	T2N0M0	29	70	M	L	T2N0M0
14	72	F	L	T1N0M0	30	86	M	L	T1N0M0
15	38	F	H	T3N0M0	31	72	M	H	T3N1M0
16	73	M	H	T3N0M0	32	76	M	L	T2N0M0

*Pt No*. patient number; *M* male; *F* female; *Grade* the World Health Organization 2004 classification; *H* high; *L* low; *Stage* the American Joint Committee on Cancer TNM classification.

### PVT1 promoted cell proliferation of bladder cancer *in vitro*

**Figure 2:**
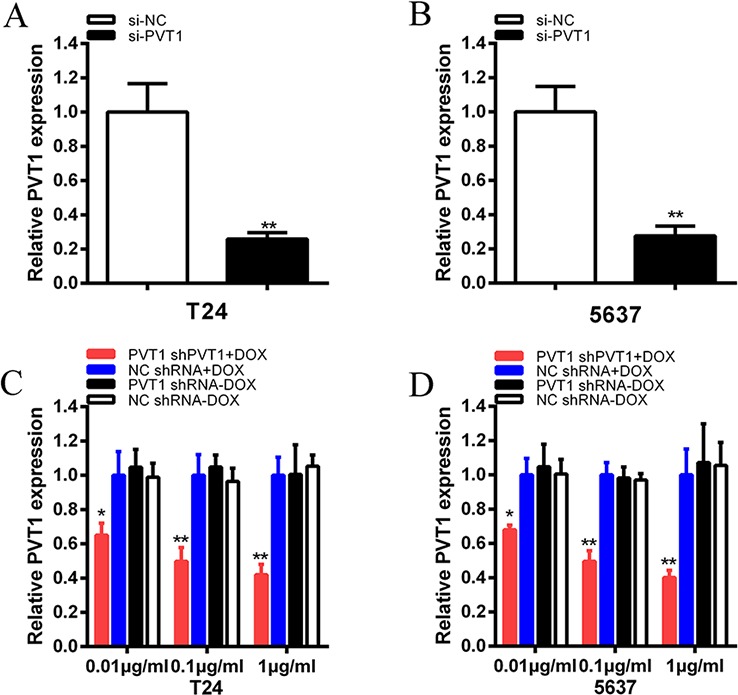
The expression levels of PVT1 were decreased after transfection of specific RNA or tetracycline inducible shRNA vectors **A, B.** The expression of PVT1 was suppressed significantly in si-PVT1 group compared with si-NC group in T24 (A) and 5637 (B) (*P* < 0.01). **C, D.** PVT1 shRNA expression was induced by doxcycline and the expression of PVT1 was down-regulated maximumly in PVT1 shRNA plus 1 μg/ml doxycycline group in T24 (C) and 5637 (D) (*P* < 0.01) and it indicated a dosage-dependent effect. Data are illustrated as mean ± SD (*P < 0.05, **P < 0.01); bar, SD.

**Figure 3:**
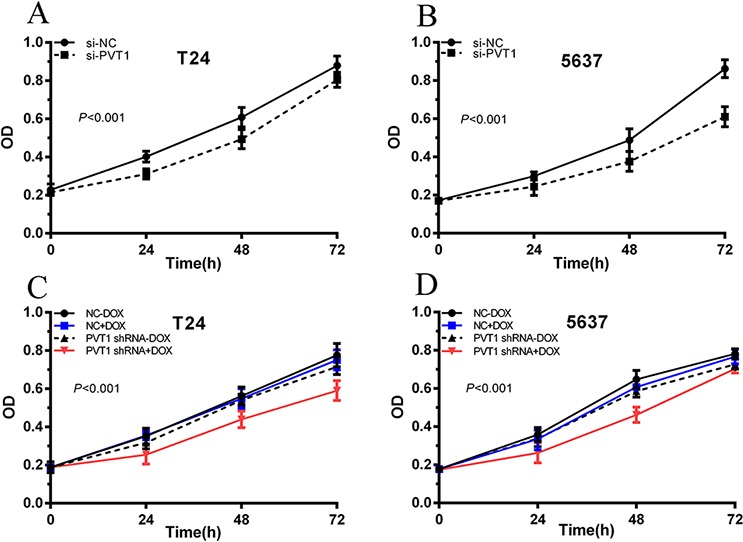
Cell proliferation was inhibited after transfection with special RNA or tetracycline-inducible shRNA vectors. CCK-8 was used to measure the cell growth ANOVA was used to analyze the data. **A, B.** After transfection with si-PVT1, cell growth inhibition was observed in T24 (A) and 5637 (B) (*P* < 0.01). **C, D.** After transfection with PVT1 shRNA plus 1 ug/ml doxycycline, cell proliferation was suppressed in T24 (C) and 5637 (D). Error bars suggest mean ± SD (*P < 0.05, **P < 0.01).

**Figure 4:**
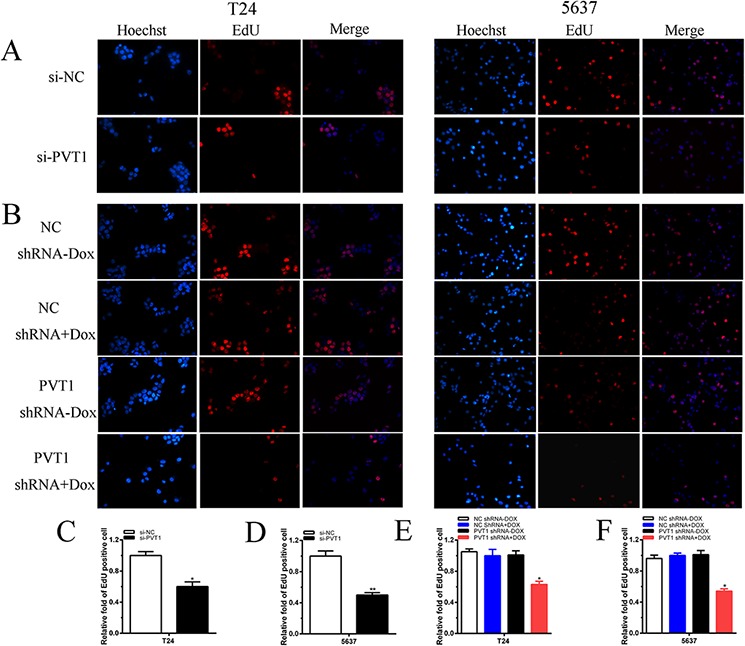
Cell growth was suppressed after transfection with special RNA or tetracycline-inducible shRNA vectors **A, B.** Representative images of EdU assay in T24 and 5637. EdU positive cells were decreased after transfected with si-PVT1 in T24 and 5637. EdU positive cells were suppressed after transfected with PVT1 shRNA plus doxycycline in T24 (A) and 5637 (B). **C, D, E, F.** EdU incorporation rate was shown as ratio of EdU positive cells relative to Hoechst 33342 positive cells. Error bars suggest mean ± SD (*P < 0.05, **P < 0.01).

To investigate functional role of PVT1 in bladder cancer cells, qRT-PCR was used to measure the relative expression level of PVT1 at 48 h post-transfection in T24 and 5637. As shown in Figure 2A and 2B, the relative expression level of PVT1 in T24 and 5637 cells were down-regulated significantly by si-PVT1 (*p* < 0.001 in two cell lines). CCK-8 assay was performed to observe whether si-PVT1 suppressed the proliferation of T24 and 5637 bladder cancer cells. The results showed that si-PVT1 suppressed cell growth significantly in bladder cancer cells (*p* < 0.001 in two cell lines) (Figure 3A and 3B). Then, a more specific and sensitive method [18, 19], EdU assay, was carried out to further detect function of PVT1 in promoting cell growth. As shown in Figure 4A, more EdU positive T24 or 5637 cells in si-NC group and less EdU positive T24 or 5637 cells in si-PVT1 group were observed after transfection of the related siRNAs. EdU assay also showed that the number of EdU positive cells in si-PVT1 group was reduced by 40% in T24 (*P* < 0.01) and decreased by 50% in 5637 (*P* < 0.01) (Figure 4C and 4D). These results indicated that PVT1 promoted cell proliferation in bladder cancer.

### PVT1 inhibited cell apoptosis of bladder cancer *in vitro*

**Figure 5:**
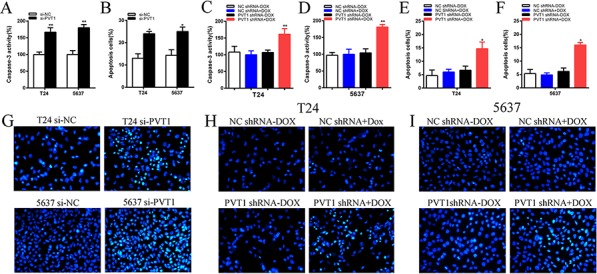
Apoptosis was induced after transfection with special RNA or tetracycline inducible shRNA vectors using ELISA and Hoechst 33258 staining assay **A.** The activity of caspase-3 was higher significantly after transfection with si-PVT1 in T24 and 5637(*P* < 0.05). **B.** Hoechst 33258 staining assays were utilized to detect apoptosis cell and more apoptosis cells were observed significantly after transfection with si-PVT1 in T24 and 5637. **C, D.** The activity of caspase-3 was higher significantly after transfection with PVT1 shRNA plus doxycycline in T24 (C) and 5637 (D) (*P* < 0.05). **E, F.** More apoptosis cells were observed significantly after transfectionwith PVT1 shRNA plus doxycycline in T24 (E) and 5637 (F). **G.** Representative images of apoptosis changes were shown after transfection with si-PVT1 in T24 and 5637. **H, I.** Representative images of apoptosis changes were presented after transfectionwith PVT1 shRNA plus doxycycline in T24 (H) and 5637 (I). Error bars suggest mean ± SD (*P < 0.05, **P < 0.01).

**Figure 6:**
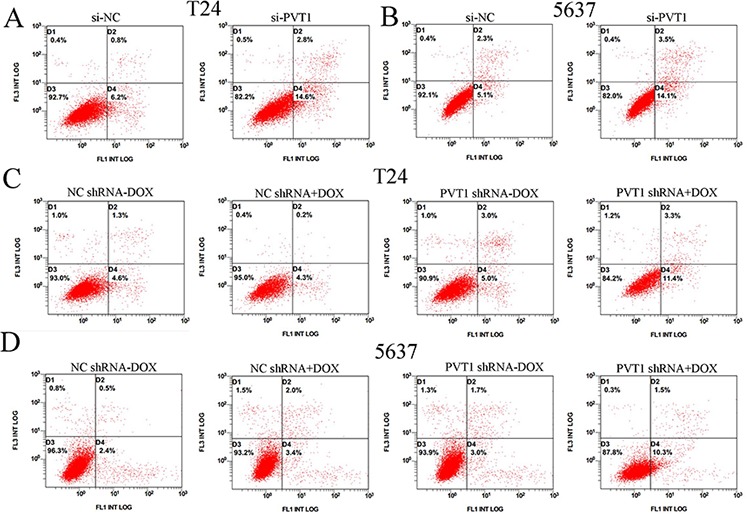
Apoptosis was induced and detected by flow cytometry analysis **A, B.** More apoptotic cells were measured in si-PVT1 group compared with si-NC group in T24 (*P* < 0.05) (A) and 5637 (*P* < 0.05) (B). **C, D.** More apoptotic cells were detected in PVT1 shRNA plus doxycycline group compared with the corresponding negative control vectors in T24 (C) (*P* < 0.05) and 5637 (D) (*P* < 0.05).

To investigate whether PVT1 suppresses apoptosis of bladder cancer cells, cells were transfected with si-PVT1 or si-NC and cell apoptosis was detected by the caspase 3 enzyme-linked immunosorbent assay (ELISA), Hoechst 33258 staining assay and flow cytometry assay. Compared with cells transfected with si-NC, the activities of caspase 3 (*P* < 0.01 in T24 cells; *P* < 0.001 in 5637 cells) and the apoptosis ratio (*P* < 0.01 in two cell lines) were increased significantly in cells transfected with the si-PVT1 (Figure 5A, 5B and 5G; Figure 6A and 6B). These results confirmed that PVT1 inhibited cell apoptosis in bladder cancer.

### Tetracycline-inducible PVT1 shRNA down regulated expression of PVT1 *in vitro*

Bladder cancer T24 and 5637 cells were cultured in 6-well plates and transfected with plasmids (2 μg) expressing either the corresponding tetracycline-inducible PVT1 shRNA or the negative control. qRT-PCR was used to detect the relative expression level of PVT1. The PVT1 shRNA induced by doxycycline could inhibit the relative expression level of PVT1 in T24 and 5637 cells significantly when added with different concentration of doxycycline (*P* < 0.01 in two cell lines) (Figure 2C and 2D). When 1 μg/ml doxycycline was added to cells transfected with PVT1 shRNA plasmids, the expression level of PVT1 in group transfected with tetracycline-inducible PVT1 shRNA was decreased by 58% in T24 (*P* < 0.01) and decreased by 60% in 5637 (*P* < 0.001). And it showed that doxycycline induced the expression of PVT1 shRNA to inhibit the expression of PVT1 in a dosage-dependent manner. As 1 μg/ml doxycycline induced the expression of PVT1 shRNA to inhibit PVT1 maximumly, we chose the concentration, 1 μg/ml, for further study.

### Tetracycline-inducible PVT1 shRNA inhibited cell proliferation *in vitro*

To detect whether cell growth was suppressed by tetracycline-inducible PVT1 shRNA, CCK8 and EdU assay were performed. CCK8 asasy suggests that compared with the negative control, tetracycline-inducible PVT1 shRNA reduced cell proliferation significantly when added with 1 ug/ml doxycycline in bladder cancer T24 and 5637 cells (*P* < 0.001 in two cell lines) (Figure 3C and 3D). EdU assay shows that less EdU positive cells in group transfected with 1 ug/ml tetracycline-inducible PVT1 shRNA in T24 and 5637 were observed (Figure 4B). EdU assay also showed that the number of EdU positive cells in group transfected with 1 ug/ml tetracycline-inducible PVT1 shRNA was decreased by 37% in T24 (*P* < 0.01) and decreased by 46% in 5637 (*P* < 0.01) (Figure 4E and 4F).

### Tetracycline-inducible PVT1 shRNA induced apoptosis of bladder cancer *in vitro*

The relative activity of caspase 3 was detected using ELISA assay. The apoptosis ratio was tested using Hoechst 33258 staining assay and flow cytometry assay. The relative activity of caspase-3 was significantly increased when cells were treated with the PVT1 shRNA induced by 1 ug/ml doxycycline (*P* < 0.01 in two cell lines) (Figure 5C and 5D). In the results of Hoechst 33258 staining assay, the number of apoptotic cells in group transfected with tetracycline-inducible PVT1 shRNA was significantly larger than group transfected with the negative control vector (*P* < 0.01 in two cell lines) (Figure 5E, 5F, 5H and 5I). Compared with the negative control group, the number of early apoptotic cells was increased significantly in tetracycline-inducible PVT1 shRNA group (Figure 6C and 6D).

## DISCUSSION

LncRNAs involve in gene regulation and extend our understanding of the biological behavior in diseases inclusive of cancers [20, 21]. As a famous oncogene in other cancer, PVT1 is located in chromosome 8q24 and is closely near MYC which promotes progression of bladder cancer [22, 23]. PVT1 is overexpressed in gastric cancer and promotes cell growth through repressing p15 and p16 [13]. Upregulation of PVT1 in hepatocellular carcinoma activates cell proliferation through stabilizing NOP2 [24]. Mouse PVT1 locus activates T lymphomagenesis through encoding several overexpressed microRNAs [25]. However, the association between PVT1 and bladder cancer is not clear.

This is the first report to illustrate the functions of PVT1 in bladder cancer. In this research, we found that the expression of non-coding RNA PVT1 was upregulated in bladder cancer tissues and cells. PVT1 was also highly correlated with histological grade and TNM stage of bladder cancer. To further understand the biological roles of PVT1 in bladder cancer, we used the CCK8, EdU assays and cell apoptosis assays to detect cell growth and apoptosis in bladder cancer cells through silencing PVT1. Cell growth arrest and increased apoptosis were observed in bladder cancer cells and these results indicated that PVT1 promoted the development of bladder cancer.

Medical synthetic biology utilizing the principles of engineering has emerged during the past several years for treating human diseases such as bladder cancer [17, 26, 27]. Inspired by the engineering principles of synthetic biology, we designed the tetracycline-inducible switch which are widely used and well known in synthetic devices to control the expression of shRNA [17, 26].

In this study, we created the shRNA device controlled by the tetracycline-inducible switch and measured its anti-cancer effects. Our data suggested PVT1 was an oncogene in bladder cancer and the tetracycline-inducible shRNA targeting PVT1 inhibited the expression of PVT1 in a dosage-dependent manner, and suppressed cell growth and induced apoptosis in bladder cancer cells. However, we should consider that this tetracycline-induced device is not absolutely efficient. Efficiency of the device may depend on the transfection efficiency. Optimizing the transfection system or packing this device as lentiviral vector may be a good choice to solve this problem.

In conclusion, PVT1 promotes progression of bladder cancer cells. Using the principles of synthetic biology, we use the synthetic platform to construct the device regulated by one of the synthetic parts, tetracycline-inducible switch, to control the expression of shRNA targeting oncogene PVT1. Our results indicate this device can quantitatively inhibit cell proliferation and induce cell apoptosis in bladder cancer cells.

## MATERIALS AND METHODS

### Cell lines and cell culture

Human bladder cancer cells (T24, 5637) used in this experiment and SV-HUC-1 cells were purchased from the Institute of Cell Research, Chinese Academic of Sciences, Shanghai, China. The T24 cells were maintained in DMEM (Invirtogen, Carlsbad, CA, USA) plus 1% antibiotics (100U/ml penicillin and100 μg/ml streptomycin sulfates) and 10% fetal bovine serum (FBS) at 37°C with an atmosphere of 5% CO_2_ in incubator. The 5637 cells were cultured in RPMI-1640 (1640) media provided with 10% FBS and 1% antibiotics in incubator (37°C, 5% CO_2_). The SV-HUC-1 cells were maintained in F12K medium (Invirtogen, Carlsbad, CA, USA) plus 1% antibiotics (100U/ml penicillin and100 μg/ml streptomycin sulfates) and 10% fetal bovine serum (FBS) at 37°C with an atmosphere of 5% CO_2_ in incubator.

### Patient samples

Thirty-two patients diagnosed with bladder cancer were included in this study. Bladder cancer tissues and matched para-cancer tissues were resected and then snap-frozen in liquid nitrogen quickly. Written formal approval was also acquired from all the patients. This research was admitted by the Institutional Review Board of Shenzhen Second People's Hospital.

### Creation of synthetic tetracycline-inducible shRNA vectors targeting PVT1

The sequences of the related tetracycline-inducible shRNA targeting PVT1 and the negative control were designed and inserted into plasmid vector PEV-Lv208 which was brought from FulenGen firm, Guangzhou, China. The tetracycline-inducible shRNA was generated with CAGCCATCATGATGGTACT.

### Transfection of cell lines

T24 and 5637 cells were transiently transfected with specific siRNA oligonucleotides, PVT1 siRNA, according to a previous study [28]. We chose the sequence, ‘CAGCCATCATGATGGTACT’, for further study. Non-specific siRNA (si-NC) and si-PVT1 were purchased from GenePharma, Suzhou, China. Bladder cancer cells were seeded in six-well plates and get 30–50% confluence before transfection. Cells were transiently transfected with si-PVT1 (100 nM) and si-NC (100 nM) by using Lipofectamine 2000 Transfection Reagent (Invitrogen, Carlsbad, CA, USA) according to the manufacturer's protocol. Plasmid Midiprep kits (Promega, Madison, USA) were used to get the plasmid vectors (PVT1 shRNA, NC shRNA) for transfection.

### RNA extraction and qRT-PCR analysis

The TRIzol reagent (Invitrogen, Grand Island, NY, USA) were used to extract total RNA from tissues or cells after transfection according to the manufacturer's instructions. Total RNA was transformed to cDNA by utilizing PrimeScript RT Reagent Kit with gDNA Eraser (Takara, Dalian, China). The primer sequences were shown: PVT1 primers [22] forward:5′-GCCCCTTCTATGGGAATCACTA-3′, reverse: 5′-GGGGCAGAGATGAAATCGTAAT-3′; GAPDH primers forward: 5′-CGCTCTCTGCTCCTCCTGTTC-3′, reverse: 5′–ATCCGTTGACTCCGACCT TCAC-3′. A standard SYBR Green PCR kit (Takara, Dalian, China) was used for quantitative PCR (qPCR) and qPCR was executed in a final reaction volume of 20 μl according to the manufacturer's instructions. GAPDH was used as the internal control and the data were normalized to the expression of GAPDH. The reactions were carried out on an ABI PRISM 7500 Fluorescent Quantitative PCR System (Applied Biosystems, Foster City, CA, USA) in triplicate. The results were analyzed by calculating the relative amount of PVT1 using the comparative ΔCt method. All experiments were carried out at least three repetitions.

### Cell proliferation assays

Cell proliferation was monitored using Cell Counting Kit-8, CCK-8 (Beyotime Institute of Biotechnology, Shanghai, China) and 5-ethynyl-20-deoxyuridine (EdU) assay kit (Ribobio, Guangzhou, China), respectively, according to the manufacturer's instructions. For CCK-8 assay, 5 × 10^3^ cells per well were seeded in a 96-well plate for 24 hours, then transiently transfected with siRNAs or plasmids. CCK-8 assay was performed according to the previous study [29]. EdU incorporation assay was carried out according to previous studies [30, 31]. All experiments were performed in triplicate.

### Caspase 3 ELISA assay

T24 and 5637 baldder cancer cells were transiently transfected with siRNA or plasmid in 12-well plates. After 48 hours, the activity of caspase 3 was detected by the caspase 3 enzyme-linked immunosorbent assay (ELISA) assay kit (Hcusabio, Wuhan, China) according to the manufacturer's protocol. All experiments were performed at least three times.

### Hoechst 33258 staining assay

Two days after transfection, the Hoechst 33258 staining kit (Life, Eugene, OR, USA) was utilized to observe the apoptotic cells induced by si-PVT1 or PVT1 shRNA plasmid [32]. Each assay was carried out at least three times.

### Flow cytometry assay

Bladder cancer T24 and 5637 cells were transiently transfected with siRNAs or plasmid vectors. 48 hours after transfection, cells were harvested and then collected. The FITC Annexin V Apoptosis Detection Kit (TransGen, Perking, China) was utilized to double stain cells with FITC-Annexin V and PI according to the manufacturer's instructions. Flow cytometry (EPICS, XL-4, Beckman, CA, USA) was utilized to observe cell apoptosis. In the graphs, cells were discriminated into dead cells, living cells, early apoptotic cells and late apoptotic cells. The ratio of early apoptotic cells was regarded as an observation index to compare the experimental group and negative group. Each experiment was done at least three times.

### Statistical analysis

All data were presented as mean ± standard deviation (SD). All statistical analyses were executed by using SPSS 20.0 software (IBM, Chicago, IL, USA). Paired samples' *t* test was used to analyze the PVT1 expression difference between bladder cancer tissues and para-cancer tissues. CCK-8assay data were analyzed by ANOVA and independent samples' *t* test was utilized to analyze other data. A two-sided value of *P* < 0.05 was considered to be statistically significant.
